# Epidemiological and etiological characteristics of 1266 patients with severe acute respiratory infection in central China, 2018–2020: a retrospective survey

**DOI:** 10.1186/s12879-024-09297-x

**Published:** 2024-04-22

**Authors:** Jin-Zhu Wang, Ding Yuan, Xiang-Hong Yang, Chang-Hua Sun, Lin-lin Hou, Yan Zhang, Hong-Xiang Xie, Yan-Xia Gao

**Affiliations:** 1 Emergency and Critical Care Center, Intensive Care Unit, Zhejiang Provincial People’s Hospital(Affiliated People’s Hospital), Hangzhou Medical College, 310014 Hangzhou, Zhejiang China; 2 Emergency Department, The First Affiliated Hospital of Zhengzhou University, 450001 Zhengzhou, Henan China; 3 Department of Clinical Laboratory Medicine, The First Affiliated Hospital of Shandong First Medical University & Shandong Provincial Qianfoshan Hospital, 250014 Jinan, Shandong China; 4 Laboratory Medicine Center, Department of Clinical Laboratory, Zhejiang Provincial People’s Hospital(Affiliated People’s Hospital), Hangzhou Medical College, 310014 Hangzhou, Zhejiang China

**Keywords:** Central China, Hospitalised patients, Severe acute respiratory infection, Respiratory pathogen, Epidemiologic characteristics

## Abstract

**Background:**

Severe acute respiratory infection (SARI), a significant global health concern, imposes a substantial disease burden. In China, there is inadequate data concerning the monitoring of respiratory pathogens, particularly bacteria, among patients with SARI. Therefore, this study aims to delineate the demographic, epidemiological, and aetiological characteristics of hospitalised SARI patients in Central China between 2018 and 2020.

**Methods:**

Eligible patients with SARI admitted to the First Affiliated Hospital of Zhengzhou University between 1 January 2018 and 31 December 2020 were included in this retrospective study. Within the first 24 h of admission, respiratory (including sputum, nasal/throat swabs, bronchoalveolar lavage fluid, thoracocentesis fluid, etc.), urine, and peripheral blood specimens were collected for viral and bacterial testing. A multiplex real-time polymerase chain reaction (PCR) diagnostic approach was used to identify human influenza virus, respiratory syncytial virus, parainfluenza virus, adenovirus, human bocavirus, human coronavirus, human metapneumovirus, and rhinovirus. Bacterial cultures of respiratory specimens were performed with a particular focus on pathogenic microorganisms, including *S. pneumoniae, S. aureus, K. pneumoniae, P. aeruginosa, Strep A, H. influenzae, A. baumannii*, and *E. coli*. In cases where bacterial culture results were negative, nucleic acid extraction was performed for PCR to assay for the above-mentioned eight bacteria, as well as *L. pneumophila* and *M. pneumoniae*. Additionally, urine specimens were exclusively used to detect Legionella antigens. Furthermore, epidemiological, demographic, and clinical data were obtained from electronic medical records.

**Results:**

The study encompassed 1266 patients, with a mean age of 54 years, among whom 61.6% (780/1266) were males, 61.4% (778/1266) were farmers, and 88.8% (1124/1266) sought medical treatment in 2020. Moreover, 80.3% (1017/1266) were housed in general wards. The most common respiratory symptoms included fever (86.8%, 1122/1266) and cough (77.8%, 986/1266). Chest imaging anomalies were detected in 62.6% (792/1266) of cases, and 58.1% (736/1266) exhibited at least one respiratory pathogen, with 28.5% (361/1266) having multiple infections. Additionally, 95.7% (1212/1266) of the patients were from Henan Province, with the highest proportion (38.3%, 486/1266) falling in the 61–80 years age bracket, predominantly (79.8%, 1010/1266) seeking medical aid in summer and autumn. Bacterial detection rate (39.0%, 495/1266) was higher than viral detection rate (36.9%, 468/1266), with the primary pathogens being influenza virus (13.8%, 175/1266), *K. pneumoniae* (10.0%, 127/1266), *S. pneumoniae* (10.0%, 127/1266), adenovirus (8.2%, 105/1266), *P. aeruginosa* (8.2%, 105/1266), *M. pneumoniae* (7.8%, 100/1266), and respiratory syncytial virus (7.7%, 98/1266). During spring and winter, there was a significant prevalence of influenza virus and human coronavirus, contrasting with the dominance of parainfluenza viruses in summer and autumn. Respiratory syncytial virus and rhinovirus exhibited higher prevalence across spring, summer, and winter. *P. aeruginosa, K. pneumoniae*, and *M. pneumoniae* were identified at similar rates throughout all seasons without distinct spikes in prevalence. However, *S. pneumoniae* showed a distinctive pattern with a prevalence that doubled during summer and winter. Moreover, the positive detection rates of various other viruses and bacteria were lower, displaying a comparatively erratic prevalence trend. Among patients admitted to the intensive care unit, the predominant nosocomial bacteria were *K. pneumoniae* (17.2%, 43/249), *A. baumannii* (13.6%, 34/249), and *P. aeruginosa* (12.4%, 31/249). Conversely, in patients from general wards, predominant pathogens included influenza virus (14.8%, 151/1017), *S. pneumoniae* (10.4%, 106/1017), and adenovirus (9.3%, 95/1017). Additionally, paediatric patients exhibited significantly higher positive detection rates for influenza virus (23.9%, 11/46) and *M. pneumoniae* (32.6%, 15/46) compared to adults and the elderly. Furthermore, adenovirus (10.0%, 67/669) and rhinovirus (6.4%, 43/669) were the primary pathogens in adults, while *K. pneumoniae* (11.8%, 65/551) and *A. baumannii* (7.1%, 39/551) prevailed among the elderly, indicating significant differences among the three age groups.

**Discussion:**

In Central China, among patients with SARI, the prevailing viruses included influenza virus, adenovirus, and respiratory syncytial virus. Among bacteria, *K. pneumoniae, S. pneumoniae, P. aeruginosa*, and *M. pneumoniae* were frequently identified, with multiple infections being very common. Additionally, there were substantial variations in the pathogen spectrum compositions concerning wards and age groups among patients. Consequently, this study holds promise in offering insights to the government for developing strategies aimed at preventing and managing respiratory infectious diseases effectively.

## Introduction

Severe acute respiratory infection (SARI) stands as a significant contributor to morbidity and mortality across all age groups, particularly affecting children, the elderly, and individuals with cardiopulmonary disease and immune deficiency [1]. Despite advances in revealing the incidence and primary causes of SARI, meeting clinical demands remains challenging [2]. The respiratory pathogens accountable for SARI encompass various viruses and bacteria, their prevalence and distribution being subject to geographic location, seasonal variations, and age groups. Therefore, comprehending the composition of the SARI-associated respiratory pathogen spectrum is an important guide for judiciously administering antivirals and antibiotics [3].

Currently, comprehensive data are scarce regarding the epidemiology of respiratory pathogens in China, with a predominant focus on paediatric populations in existing studies. A comparative analysis in China explored viral test outcomes in hospitalised children with SARI in Beijing and Shanghai, unveiling distinct age-specific and seasonal distribution patterns of viral infections between the two cities. Rhinovirus (RV) and enterovirus emerged as the most prevalent, followed by respiratory syncytial virus (RSV), human bocavirus (HBoV), adenovirus (ADV), human coronavirus (HCoV), and influenza viruses [4]. A 3-year surveillance in Wuhan, China, highlighted that the detection rate of respiratory viruses among children with SARI peaked in January and December decreasing significantly in February and August. Influenza virus was detected at the highest rate before, and RSV after, the coronavirus disease 2019 (COVID-19) pandemic [5]. These findings significantly contributed to understanding the composition of the respiratory pathogen spectrums linked to SARI in children. In a pilot hospital-based surveillance in Jinshan, Shanghai, examining etiological and epidemiological characteristics of SARI caused by multiple viruses and Mycoplasma pneumoniae in adult patients, the findings revealed that among 397 patients enrolled, 250 (63.0%) tested positive for at least one pathogen, with 52 (13.1%) patients testing positive for multiple pathogens. Mycoplasma pneumoniae (23.9%), influenza virus (21.4%), and ADV (11.6%) were the most frequently identified pathogens [6].

In the recent surge of hospitalised patients with SARI, bacterial infections have emerged as a noteworthy contributor alongside the common viral infections and a significant proportion of patients have mixed infections [7–9]. Currently, there is a dearth of studies concentrating specifically on respiratory pathogens, particularly bacterial infections, among individuals with SARI in China. This study, encompassing 1266 patients with SARI admitted to the First Affiliated Hospital of Zhengzhou University from 2018 to 2020, aims to delineate the demographic, epidemiological, and aetiological characteristics of these patients in Central China.

## Materials and methods

### Participants

Henan Province, situated in Central China, boasts a population exceeding 100 million. The First Affiliated Hospital of Zhengzhou University, located in the province’s capital, Zhengzhou, stands as a large healthcare facility accommodating up to 10,000 beds. Each year, it admits a substantial number of patients with respiratory infections from within and outside the province. Additionally, it serves as a national surveillance sentinel for monitoring respiratory pathogens.

**Fig. 1:**
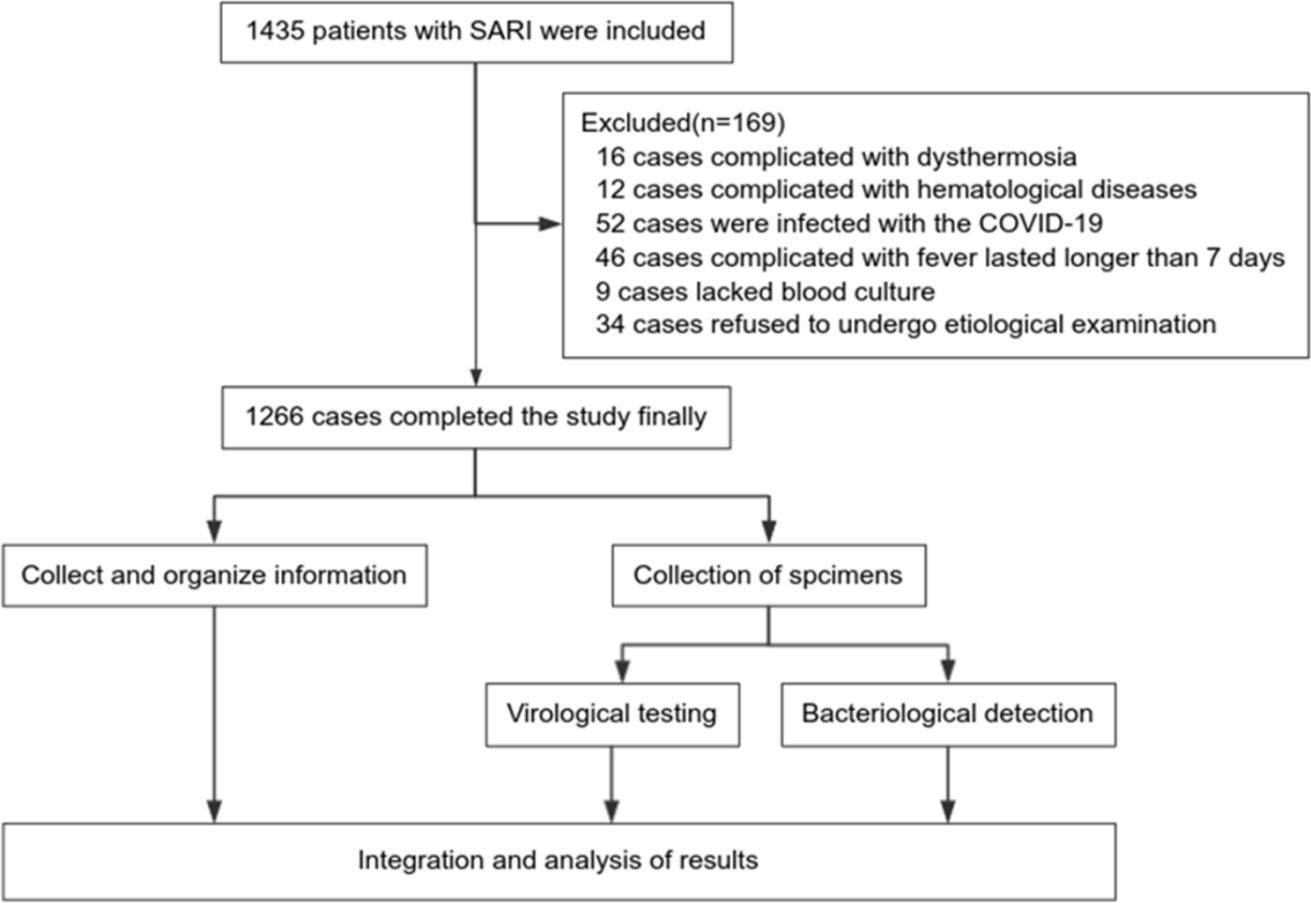
Study flow chart

Patients meeting the criteria for SARI and admitted to the First Affiliated Hospital of Zhengzhou University from 1 January 2018 to 31 December 2020 were retrospectively enrolled. SARI diagnosis aligned with the definition outlined by the World Health Organisation [10]: the presence of acute respiratory infection symptoms, along with a history of fever (≥ 38 °C), new onset of cough or worsening of pre-existing cough, necessitating hospitalisation within the preceding 10 days. The exclusion criteria encompassed conditions such as thermoregulatory and haematological system disorders, duration from fever onset to visit exceeding > 7 days, and refusal to undergo etiological examinations, as demonstrated in Fig. 1.

Patients within this study underwent categorisation based on age groups: individuals ≤ 14 years were identified as paediatric patients, those aged over 14 years and ≤ 60 years were classified as adult patients, and those aged > 60 years were categorised as elderly patients.

### Study methods

#### Collection of clinical data

Upon admission, comprehensive demographic and clinical data were recorded for all patients. This encompassed details such as age, sex, occupation, date of visit, patient distribution (intensive care unit [ICU], respiratory ward, internal medicine ward, surgical ward, paediatric ward, and emergency ward), duration from fever onset to hospital visit, primary clinical symptoms, history of chronic diseases, pre-admission antibiotic usage, chest imaging findings, and infection type, and other pertinent factors.

#### Collection, transportation, and storage of specimens

Within 24 h of admission (prior to treatment initiation), experienced nurses collected respiratory and blood specimens following standard protocols for viral and bacterial testing. For viral tests, nasal/throat swabs were mandatory, while, if feasible, sputum, bronchoalveolar lavage fluid (BALF), thoracocentesis fluid, and peripheral blood were also obtained. Regarding bacterial specimens, peripheral blood, sputum, and urine were mandatory, and BALF and thoracocentesis fluid were collected if conditions permitted. Respiratory physicians performed BALF collection through fibreoptic bronchoscopy lavage when necessary, while thoracocentesis fluid collection was performed based on the condition of the patients with pleural effusion. Blood culture flasks needed to be submitted within 24 h and stored at room temperature (15–30 °C) before submission, while other specimens were stored at 4–8℃ before submission. During specimen transportation, blood culture flasks were stored at 15–30℃, whereas other specimens were transported at 4–8 ℃. All specimens were promptly tested for viruses and bacteria upon arrival at the testing laboratory of the First Affiliated Hospital of Zhengzhou University. Viral test specimens that could be examined within 24 h were stored at 4℃, while those requiring longer storage were kept at ≤-70℃. Bacterial culture specimens were isolated immediately to avoid repeated freeze-thaw cycles.

#### Detection of pathogens

##### Bacterial detection process

The collected respiratory specimens (sputum, BALF, and thoracocentesis fluid) underwent processing and were inoculated into blood agar, MacConkey agar, and chocolate agar culture media. Concurrently, peripheral blood specimens were introduced into blood culture flasks upon collection. All media were then incubated in a 5–10% carbon dioxide environment at 35 °C, maintaining a humid setting for 18–24 h. Subsequently, suspected bacteria displaying distinctive bacterial colony morphology were selected for isolation into pure culture. The eight primary pathogenic microorganisms encompassed *Streptococcus pneumoniae(S. pneumoniae), Staphylococcus aureus(S.aureus), Klebsiella pneumoniae(K.pneumoniae), Pseudomonas aeruginosa(P. aeruginosa), Group A β Streptococcus(Strep A), Haemophilus influenzae(H. influenzae), Acinetobacter baumannii(A. baumannii), and Escherichia coli(E.coli)*. In instances where bacterial culture results were negative, nucleic acid extraction was performed following the prescribed protocol for PCR assay. The commercial kit used for bacterial DNA extraction and PCR detection was Genomic DNA Extraction Kits (Thermo Fisher Scientific, Shanghai, China). This process targeted the above-mentioned eight bacteria in addition to Legionella pneumophila*(L. pneumophila)* and Mycoplasma pneumoniae(*M. pneumoniae*). Urine specimens were exclusively used for Legionella antigen detection, using kits provided by HEPENGBIO (Shanghai, China).

##### Viral detection process

The respiratory specimens (nasal/throat swabs, sputum, BALF, and thoracocentesis fluid) obtained were packaged and processed for nucleic acid extraction. Using the QIAamp Viral RNA/DNA Mini-Kit (Qiagen, Hilden, Germany) according to the manufacturer’s instructions, viral ribonucleic acid (RNA) and deoxyribonucleic acid (DNA) were extracted from 200 µL samples. RNA viruses were identified via reverse transcription-PCR, while DNA viruses were detected using the PCR method. The FTD Respiratory Pathogens 21 (Fast Track Diagnostics) multiplex real-time PCR (Premedical Laboratories, Beijing, China) diagnostic approach was used to detect human influenza virus, RSV, parainfluenza virus (PIV), ADV, HBoV, HCoV, human metapneumovirus, and RV. Specimens testing positive for nucleic acids underwent further analysis via virus isolation and variation assessment. In certain instances, peripheral blood specimens were chosen for antibody testing to suspected viruses (if necessary). Specimens negative for nucleic acids or peripheral blood viral antibodies were further analysed for unknown pathogens (if necessary).

### Statistical methods

Statistical analyses were conducted using SPSS 24.0 software. Normally distributed measurement data are presented as the mean ± standard deviation (x ± s), and non-normally distributed data are presented as the median and interquartile range. Categorical data are presented as the number of cases and percentage, and compared using the χ^2^ test or Fisher’s exact test. A P-value of < 0.05 was considered statistically significant.

## Results

### General clinical data

This study encompassed 1266 patients, with a mean age of 54 years; among them, 64.4% (811/1266) were classified as adult patients. The cohort comprised 61.6% (780/1266) males and 61.4% (778/1266) individuals were farmers. A significant majority, 88.8% (1124/1266), sought medical treatment in 2020, with the majority (79.8%, 1010/1266) seeking treatment during summer and autumn. Additionally, 80.3% (1017/1266) were admitted to general wards. Common respiratory symptoms included fever (86.8%, 1122/1266) and cough (77.8%, 986/1266). Anomalies were detected in chest imaging studies for 62.6% (792/1266) of the patients, while 10.7% (135/1266) had received antibiotic therapy before admission. Moreover, 65.4% (828/1266) underwent BALF collection. Among the cohort, 58.1% (736/1266) had at least one identified respiratory pathogen, as detailed in Table 1.

**Table 1:** Demographic and clinical characteristics of 1266 patients with severe acute respiratory infection

Patient characteristics	All patients (*n* = 1266)
Age [years, x ± s]	54 ± 18
Age distribution [n (%)]	
≤ 14 years old	46 (3.6)
15–60 years old	669 (52.8)
> 60 years old	551 (43.6)
Sex [n (%)]	
Male	780 (61.6)
Female	486 (38.4)
Geographic distribution [n (%)]	
Henan Province	1212 (95.7)
Other provinces	54 (4.3)
Occupation type [n (%)]	
Farmer	778 (61.4)
Retiree	97 (7.6)
Student	83 (6.6)
Worker	92 (7.2)
Cadres and office clerk	186 (14.6)
Others	5 (0.4)
Unknown	25 (1.9)
Year of visit [n (%)]	
2018	39 (3.1)
2019	103 (8.1)
2020	1124 (88.8)
Season at the time of visit [n (%)]	
Spring	120 (9.5)
Summer	402 (31.8)
Autumn	608 (48.0)
Winter	136 (10.7)
Patient distribution [n (%)]	
Emergency ward	421 (33.2)
Respiratory ward	420 (33.2)
Internal medicine ward	83 (6.6)
Surgical ward	66 (5.2)
Paediatric ward	27 (2.1)
ICU	249 (19.7)
Time from onset to treatment [d, x ± s]	2 ± 1
Major respiratory symptoms and signs [n (%)]	
Fever	1122 (86.8)
Cough	986 (77.8)
Expectoration	462 (36.4)
Shortness of breath	253 (19.9)
Difficulty breathing	618 (48.8)
Chest pain	119 (9.4)
Pulmonary rales	224 (17.7)
Concomitant symptoms [n (%)]	
Headache	74 (5.8)
Weakness	152 (12.0)
Abdominal pain	65 (5.1)
Nausea	30 (2.4)
Dizziness	38 (3.0)
Arthralgia	22 (1.7)
Chest imaging [n (%)]	
Unremarkable	21 (1.6)
Pulmonary exudation	144 (11.4)
Pulmonary hyperinflation	130 (10.3)
Pleural effusions	419 (33.1)
Lymphadenectasis	99 (7.8)
Not checked	290 (22.9)
Chronic medical conditions [n (%)]	
Heart disease	107 (8.4)
Hypertension	247 (19.5)
Diabetes mellitus	111 (8.7)
Chronic respiratory disease	21 (1.6)
CKD	12 (0.9)
Use of antibiotics before admission [n (%)]	
Yes	135 (10.7)
No	895 (70.7)
Unclear	236 (18.6)
Special respiratory specimens [n (%)]	
BALF	828 (65.4)
Thoracentesis fluid	104 (8.2)
Single infection [n (%)]	385 (30.4)
Multiple infections [n (%)]	
Two pathogens	208 (16.4)
Three pathogens	113 (8.9)
Four or more pathogens	40 (3.1)

ICU, intensive care unit; CKD, chronic kidney disease; BALF, bronchoalveolar lavage fluid

### Epidemiological characteristics of 1266 patients with SARI

**Fig. 2:**
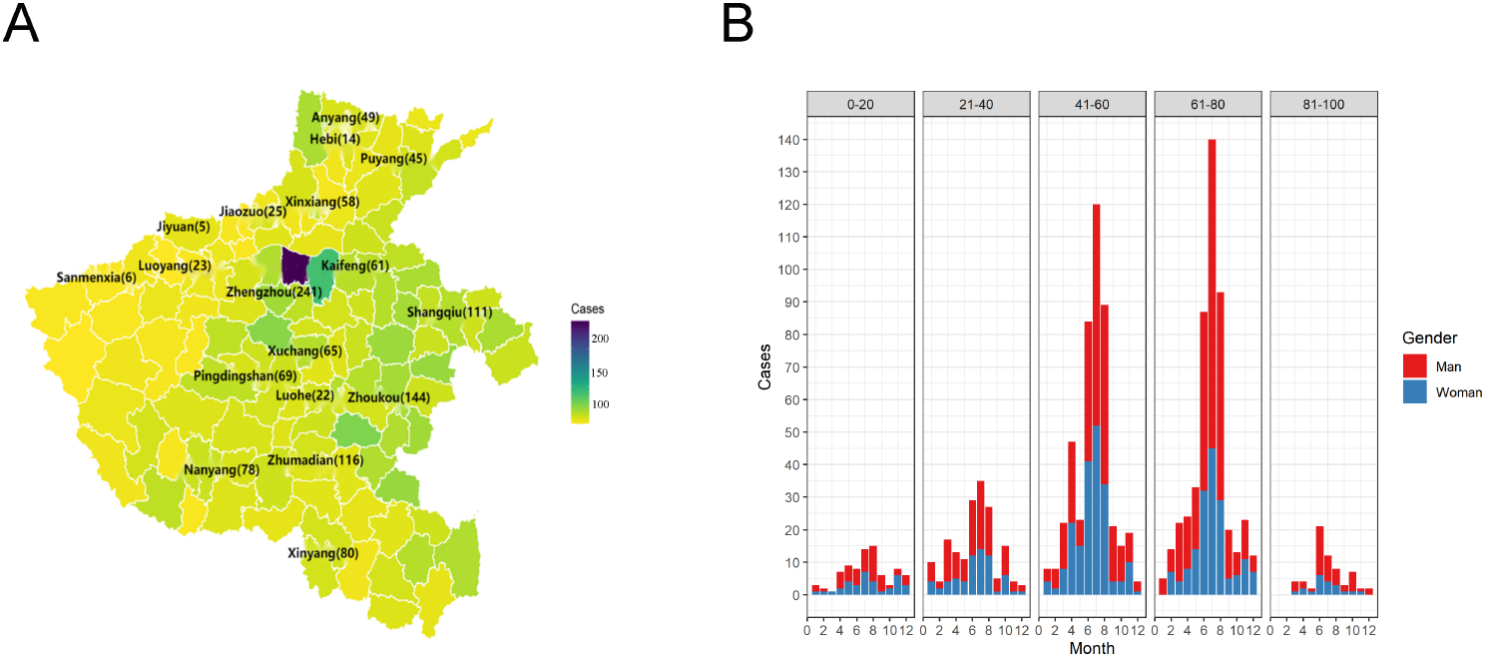
Epidemiological characteristics of 1266 patients with severe acute respiratory infection. Geographic distribution (**A**) distribution of patients according to sex and age by the month of visit (**B**)

Furthermore, 95.7% (1212/1266) of the participants were from Henan Province, predominantly residing in and around Zhengzhou. Notably, there was a higher representation of patients from the eastern region in comparison to the western part of Henan Province (A). Males (61.6%, 780/1266) constituted the majority, while individuals aged 61–80 years (38.3%, 486/1266) accounted for the largest demographic group, followed by those aged 41–60 years (36.3%, 460/1266). Seeking medical treatment peaked during autumn (48.0%, 608/1266), followed by summer (31.8%, 402/1266) (B), as presented in Fig. 2.

### Analysis of the respiratory pathogen spectrum of 1266 patients with SARI

**Fig. 3:**
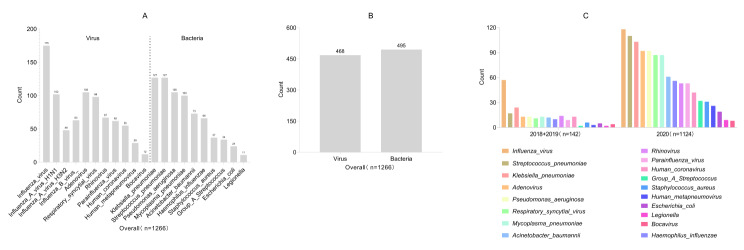
Respiratory pathogen spectrum of 1266 patients with severe acute respiratory infection. Overall pathogen distribution (**A**) comparison of the number of patients with positive bacterial and viral tests (**B**) pathogen distribution across different years (**C**)

Most patients (88.8%, 1124/1266) sought medical treatment in 2020, and the pathogen spectrums were similar in 2018, 2019, and 2020 (C). Among the 1266 patients with SARI, the top five viruses identified were influenza virus (13.8%, 175/1266), ADV (8.2%. 105/1266), RSV (7.7%, 98/1266), RV (5.2%, 67/1266), and PIV (4.8%, 62/1266). The primary subtypes of influenza virus were influenza A virus H_1_N_1_(8.0%), influenza B virus(4.9%), and influenza A virus H_3_N_2_(3.7%).The top five bacteria identified were *K. pneumoniae* (10.0%, 127/1266), *S. pneumoniae* (10.0%, 127/1266), *P. aeruginosa* (8.2%, 105/1266), *M. pneumoniae* (7.8%, 100/1266), *A. baumannii* (5.7%, 73/1266), and *H. influenzae* (5.2%, 66/1266) (A). The overall bacterial detection rate stood higher at 39.0% (495/1266) compared to the viral detection rate of 36.9% (468/1266) (B). The predominant pathogen spectrum comprised influenza virus (13.8%, 175/1266), followed by *K. pneumoniae* (10.0%, 127/1266), *S. pneumoniae* (10.0%, 127/1266), ADV (8.2%, 105/1266), *P. aeruginosa* (8.2%, 105/1266), *M. pneumoniae* (7.8%, 100/1266), and RSV (7.7%, 98/1266) (A), as depicted in Fig. 3.

### Differences in the pathogen spectrum among patients in different years of visits

**Fig. 4:**
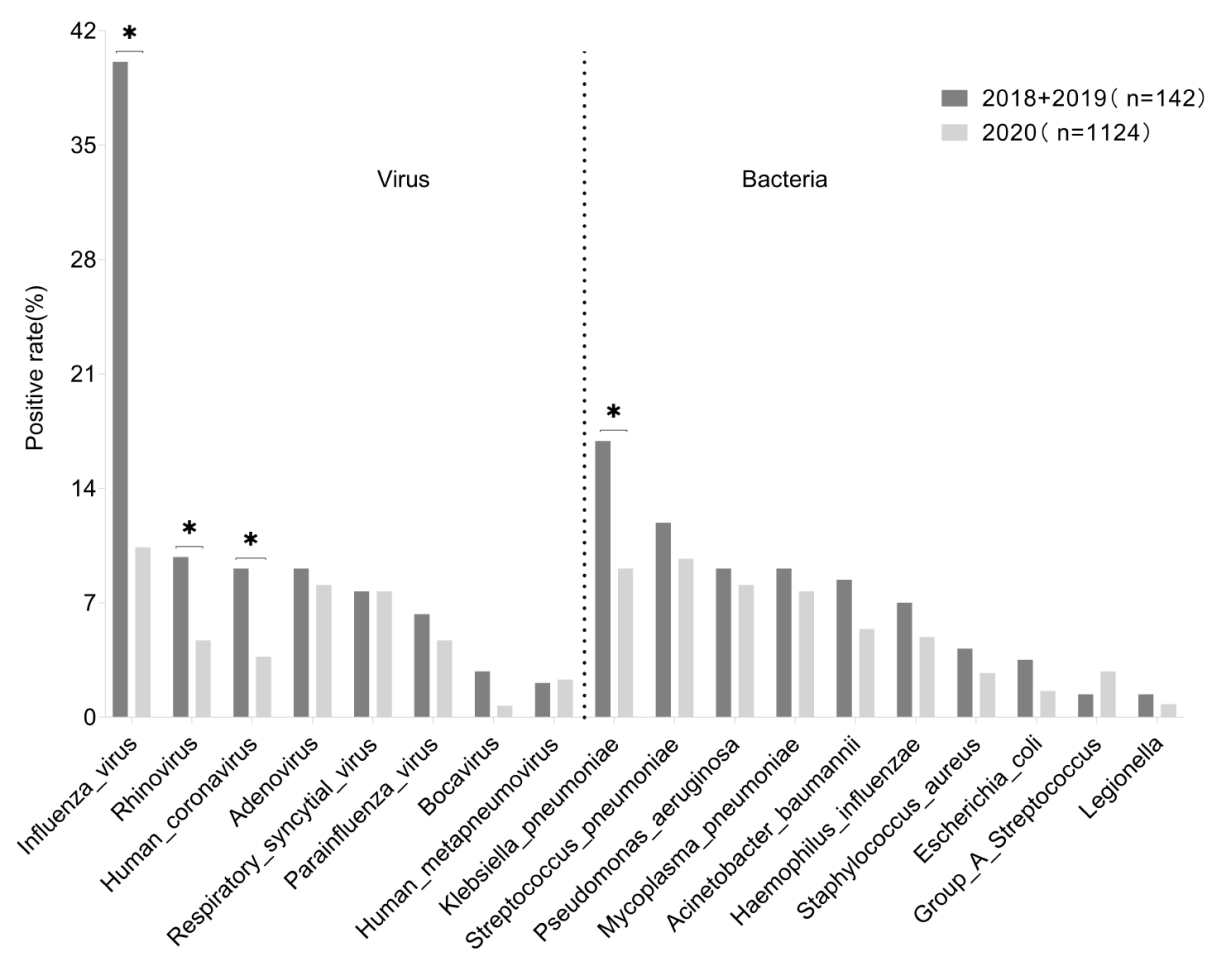
Differences in the pathogen spectrum among patients in different years of visits.For each pathogen, the positive rate on the Y-axis is the number of patients who tested positive for respiratory pathogen in that year divided by the number of patients in that year

Dividing patients into two groups, 2018 and 2019, and comparing them with the pathogen spectrum of patients in 2020. It was found that the positive rates of influenza virus, rhinovirus, human coronaviruses, and *K. pneumoniae* in the 2020 group were significantly lower than those in the 2018 and 2019 groups, and the differences were statistically significant (all *P* < 0.05). The detection rates of adenovirus, respiratory syncytial virus, bocavirus, *S. pneumoniae*, *P. aeruginosa*, *M. pneumoniae*, *A. baumannii*, *H. influenzae*, *S. aureus*, *E. coli*, and *L. pneumophila* in the 2020 group were also lower than those in the 2018 and 2019 groups, but no statistical differences were observed (all *P* > 0.05), as presented in Fig. 4.

### Respiratory viral detection in patients with SARI seeking medical treatment in different seasons

**Fig. 5:**
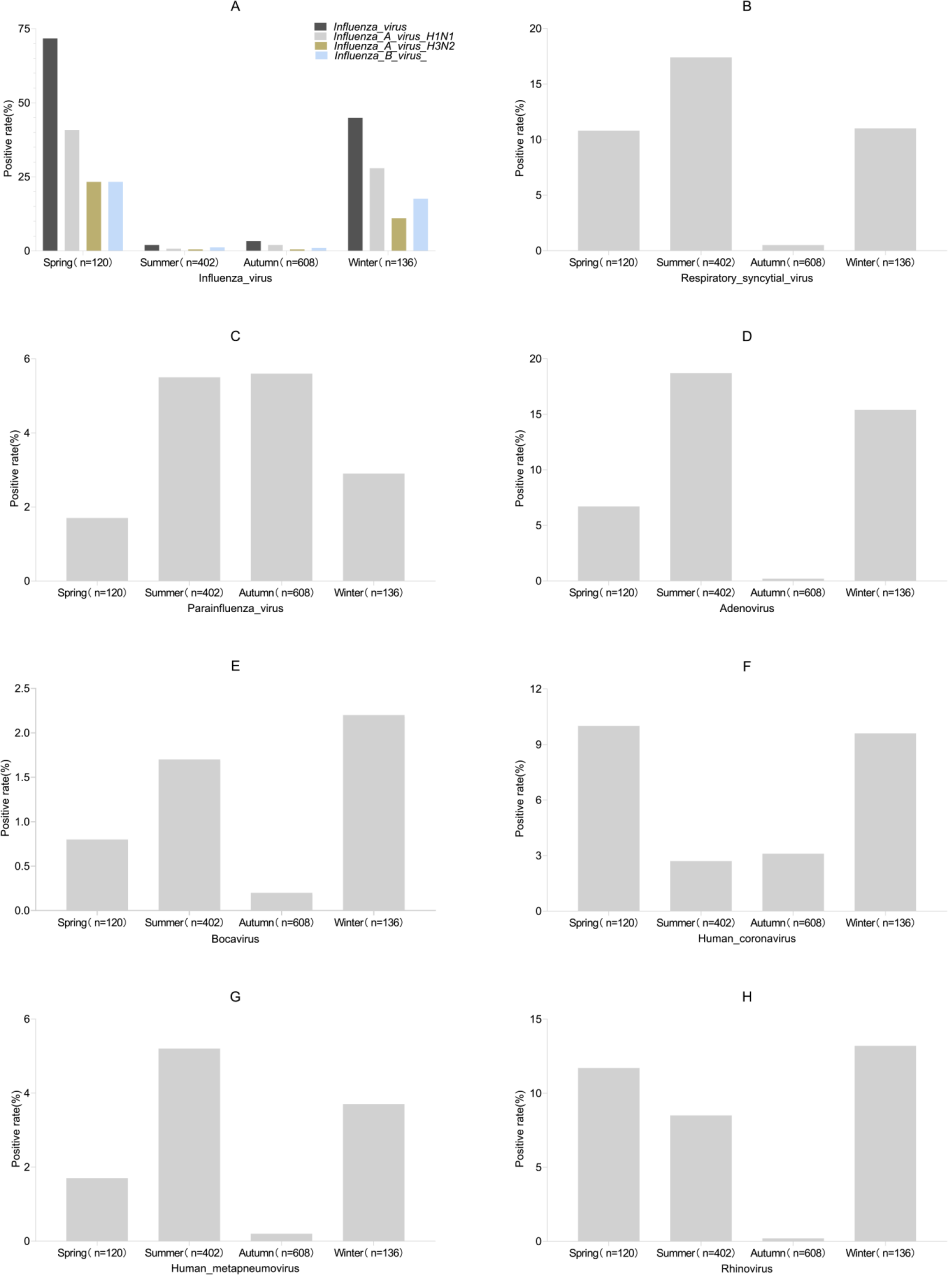
Detection of respiratory viruses in patients with severe acute respiratory infection seeking medical treatment in different seasons. Each image illustrates the seasonal distribution of the pathogen. For each pathogen, the positive rate on the Y-axis is the number of patients who tested positive for respiratory viruses in that season divided by the number of patients in that season. Influenza virus (**A**) respiratory syncytial virus (**B**) parainfluenza virus (**C**) adenovirus (**D**) human bocavirus (**E**) human coronavirus (**F**) human metapneumovirus (**G**) rhinovirus (**H**)

Influenza virus (A) and HCoV (F) exhibited significant prevalence during spring and winter. Particularly notable was the high detection rate of influenza virus at 71.6% (86/120) in spring and 44.8% (61/136) in winter. Conversely, their prevalence trend appeared relatively sporadic in other seasons. PIV (C) demonstrated predominant prevalence in summer and autumn, recording positive rates of 5.5% (22/402) and 5.6% (34/608), respectively. RSV (B) and RV (H), with notably higher positive detection rates, were more frequently observed in spring, summer, and winter, and less common in autumn. On the other hand, several other viruses exhibited lower detection rates and relatively sporadic prevalence trends (D, E, and G), as presented in Fig. 5.

### Respiratory bacterial detection in patients with SARI seeking medical treatment in different seasons

**Fig. 6:**
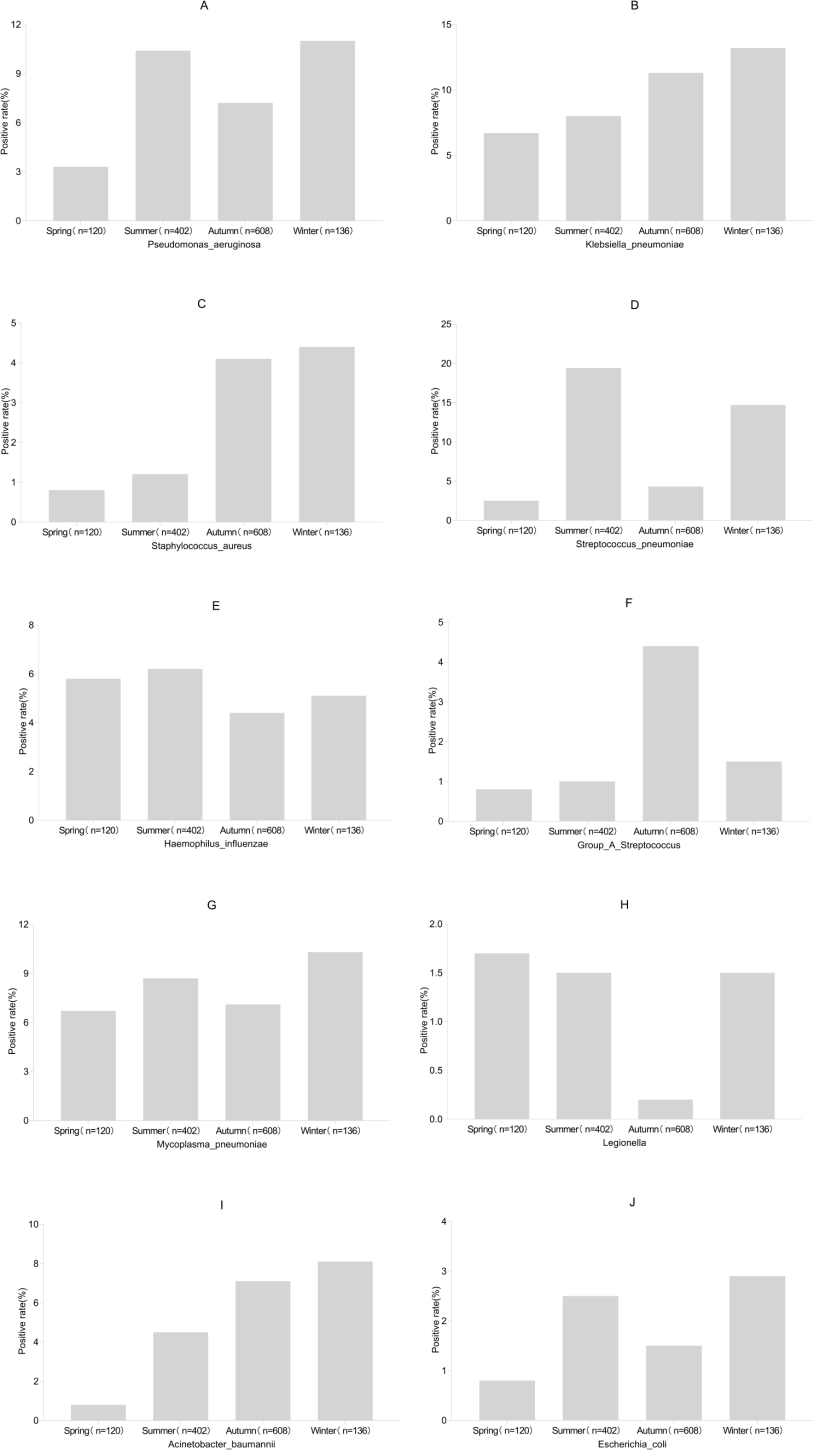
Detection of respiratory bacteria in patients with severe acute respiratory infection seeking medical treatment in different seasons. Each image illustrates the seasonal distribution of the pathogens. For each pathogen, the positive rate on the Y-axis is the number of patients who tested positive for respiratory bacteria in that season divided by the number of patients in that season. Pseudomonas aeruginosa (**A**) Klebsiella pneumoniae (**B**) Staphylococcus aureus (**C**) Streptococcus pneumoniae (**D**) Haemophilus influenzae (**E**) Group A haemolytic streptococcus (**F**) Mycoplasma pneumoniae (**G**) Legionella (**H**) Acinetobacter baumannii (**I**); Escherichia coli (**J**)

*P. aeruginosa* (A), *K. pneumoniae* (B), and *M. pneumoniae* (G), exhibiting higher positive detection rates, maintained similar rates across all seasons. In contrast, *S. pneumoniae* (D) displayed a “double peak” prevalence in summer and winter. The positive detection rates of several other bacteria were lower, and their prevalence trend was relatively sporadic (C, E, F, H, I, and J), as presented in Fig. 6.

### Differences in respiratory pathogen spectrums between patients with SARI in the ICU and those in general wards

**Fig. 7:**
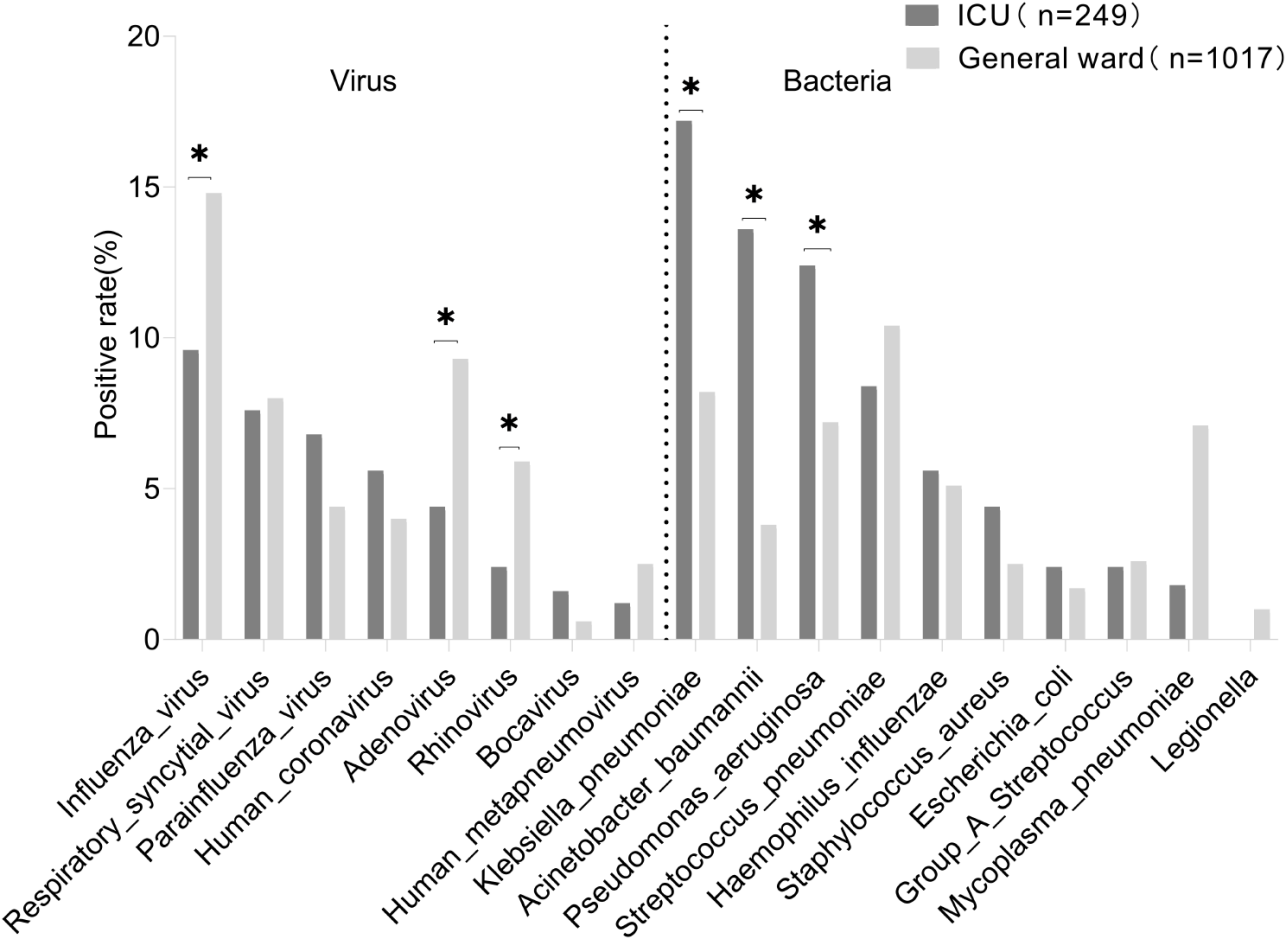
Differences in respiratory pathogen spectrums between patients with severe acute respiratory infection admitted to the intensive care unit and those in general wards. The positive rate on the Y-axis is the number of patients who tested positive for respiratory pathogens divided by the total number of patients. Statistical significance in terms of the detection rate between the two groups was set at *P* < 0.05 (marked with an asterisk)

Among the 249 patients admitted to the ICU, the top five identified pathogen spectrums comprised *K. pneumoniae* (17.2%, 43/249), *A. baumannii* (13.6%, 34/249), *P. aeruginosa* (12.4%, 31/249), influenza virus (9.6%, 24/249), and *S. pneumoniae* (8.4%, 21/249). Conversely, among the 1017 patients in general wards, the top five pathogen spectrums comprised influenza virus (14.8%, 151/1017), *S. pneumoniae* (10.4%, 106/1017), ADV (9.3%, 95/1017), *K. pneumoniae* (8.2%, 84/1017), and RSV (8.0%, 82/1017). Moreover, patients admitted to the ICU exhibited significantly higher positive detection rates than patients in general wards for K. pneumoniae (17.2% vs. 8.2%), *A. baumannii* (13.6% vs. 3.8%), *P. aeruginosa* (12.4% vs. 7.2%), *S. aureus* (4.4% vs. 2.5%), HCoV (5.6% vs. 4.4%), and PIV (6.8% vs. 4.4%). Conversely, patients admitted to the ICU had lower positive detection rates of influenza virus (9.6% vs. 14.8%), ADV (4.4% vs. 9.3%), RV (2.4% vs. 5.9%), and *M. pneumoniae* (1.8% vs. 7.1%), as depicted in Fig. 7.

### Differences in respiratory pathogen spectrums among patients with SARI across different age groups

**Fig. 8:**
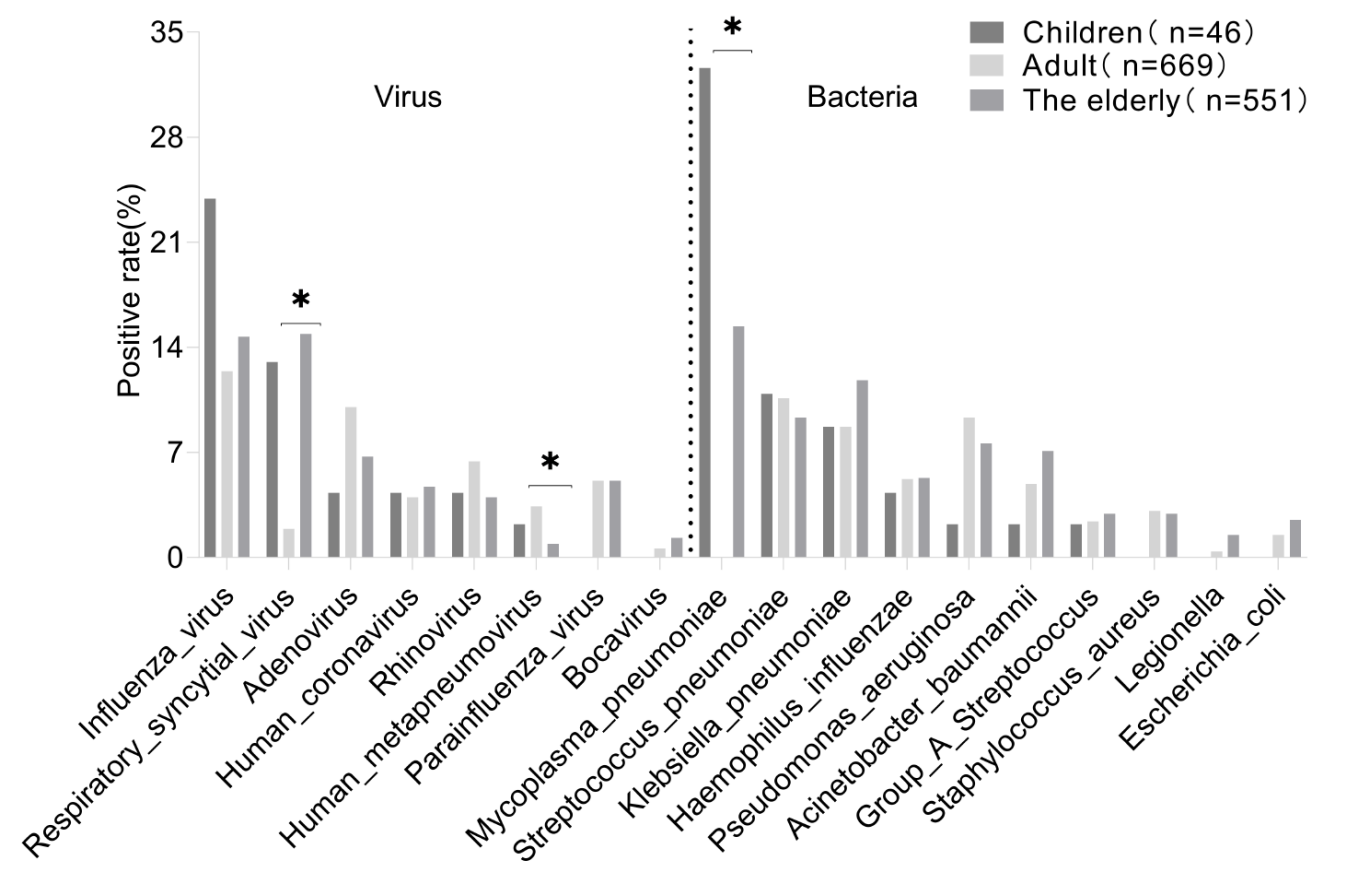
Differences in the respiratory pathogen spectrums among patients with severe acute respiratory infection across different age groups. The positive rate on the Y-axis is the number of patients who test positive for respiratory pathogens divided by the total number of patients. Statistical significance in terms of the detection rate among the three groups was set at *P* < 0.05 (marked with an asterisk)

The positive detection rates of influenza virus and *M. pneumoniae* reached as high as 23.9% (11/46) and 32.6% (15/46), respectively, in paediatric patients, significantly surpassing those observed in adult and elderly patients. Moreover, among adults, ADV (10.0%, 67/669) and RV (6.4%, 43/669) emerged as the primary pathogens, whereas in the elderly, *K. pneumoniae* (11.8%, 65/551) and *A. baumannii* (7.1%, 39/551) were predominant, exhibiting significant differences among the three age groups. Notably, there were no significant differences observed in the positive detection rates of other viruses and bacteria across the age groups, as depicted in Fig. 8.

## Discussion

Routine surveillance of the SARI-associated respiratory pathogen spectrum and epidemiological characteristics within a country or region serves as an important guide for developing appropriate prevention and control strategies. This study described the epidemiological characteristics of common viruses and bacteria responsible for SARI in Henan Province over 3 years (2018–2020). The findings indicated a substantial influx of patients (79.8%) seeking medical treatment during summer and autumn, contrasting with fewer visits in winter, possibly linked to the COVID-19 pandemic at the end of 2019. During this period, comprehensive government-imposed measures aimed to curb population movement [11]. Moreover, a predominant 95.7% hailed from Henan Province, particularly from the vicinity of Zhengzhou. Moreover, the higher patient count from the eastern part of Henan Province might stem from the limited medical resources, leading patients to opt for treatment in Zhengzhou, abundant in medical facilities. This underscores regional disparities in resource distribution. Additionally, individuals aged 61–80 years constituted a significant proportion and faced heightened susceptibility to respiratory infections. The majority (80.3%) were from general wards, with 13.2% requiring ICU care, Notably comprising elderly individuals with underlying health conditions or compromised immune systems—consistent with prevailing research [12]. Furthermore, fever (86.8%) and cough (77.8%) emerged as the most prevalent clinical symptoms, aligning with previous findings [6, 13]. Chest imaging revealed anomalies in 62.6% of cases, suggesting a likelihood of over half of the patients eventually receiving a diagnosis of community-acquired pneumonia.

Although the main consultation time for patients in this study is in 2020, the pathogen spectrum in these three years (2018–2020) shows a similar trend. The premise for obtaining this result is that patients infected with COVID-19 are excluded from this study. As we all know, the COVID-19 pandemic has caused a huge disease and economic burden worldwide [14, 15]. Wearing masks for personal protection and maintaining a certain social distance has become an important measure for “epidemic prevention,” and this recommendation has been deeply rooted in the public consciousness [16]. Our study found that the positive rates of influenza viruses, rhinoviruses, human coronaviruses, and *K. pneumoniae* detection in the 2020 group were significantly lower than those in the 2018 and 2019 groups, and the differences were statistically significant (all *P* < 0.05). In addition, the detection rates of adenoviruses, respiratory syncytial viruses, bocaviruses, *S. pneumoniae, P. aeruginosa, M. pneumoniae, A. baumannii, H. influenzae, S. aureus, E. coli, and L. pneumophila* in the 2020 group were also lower than those in the 2018 and 2019 groups, but no statistical differences were shown (all *P* > 0.05). This indicates that standardized mask wearing, as a non-pharmaceutical intervention measure, is not only effective in reducing COVID-19 virus infections but also effective in preventing the transmission of other pathogens.

While most patients sought medical treatment in 2020, the pathogen spectrums observed over 3 years (2018–2020) exhibited a consistent trend, contingent upon the exclusion of individuals infected with COVID-19 from this study. Moreover, 58.1% (736/1266) of the patient cohort had at least one identifiable respiratory pathogen, with the bacterial detection rate (39.0%) marginally surpassing the viral detection rate (36.9%), contrary to findings in other studies. Furthermore, the surveillance focused on monitoring the incidence of bacterial infections in SARI. The higher bacterial positive detection rates observed in this study may be attributed to the fact that up to 65.4% of the patients underwent BALF collection. Previous studies have demonstrated that BALF, being a lower respiratory specimen, exhibits significantly higher rates of positive bacterial culture compared to upper respiratory specimens, particularly in cases of life-threatening lung infections. In such cases, employing metagenome next-generation sequencing can expedite the identification of pathogens [17–19].

The study identified the top five viruses as influenza virus (13.8%), ADV (8.2%), RSV (7.7%), RV (5.2%), and PIV (4.8%). Further analysis of influenza virus subtypes revealed influenza A virus H_1_N_1_, influenza A virus H_3_N_2,_ and influenza B virus as the primary subtypes, prevalent during winter and spring, aligning with the previous surveillance in Hubei [20, 21]. This emphasises the importance of influenza vaccination, particularly for high-risk groups such as infants, young children, and elderly individuals with underlying diseases, as a preventive measure. HCoV exhibited a significant prevalence trend in spring and winter, while PIV predominated in summer and autumn. Additionally, RSV and RV, with higher positive detection rates, were more prevalent in spring, summer, and winter but less common in autumn. Conversely, several other viruses exhibited relatively low detection rates and sporadic prevalence trends, a consistent epidemiological feature observed in previous reports [4]. Of the 1266 patients, 28.5% presented with multiple infections, primarily a combination of bacterial and viral infections. Similar patterns have been reported in previous studies [22]. This could be attributed to viral infections disrupting airway tissues and function, thereby increasing susceptibility to bacterial invasion [23, 24].

The top five bacteria identified in this study were *K. pneumoniae* (10.0%), *S. pneumoniae* (10.0%), *P. aeruginosa* (8.2%), *M. pneumoniae* (7.8%), *A. baumannii* (5.7%), and *H. influenzae* (5.2%). Notably, *S. pneumoniae, M. pneumoniae*, and *H. influenzae* are commonly associated with community-acquired pneumonia, whereas *K. pneumoniae, P. aeruginosa*, and *A. baumannii* are frequently associated with hospital-acquired pneumonia. These findings align with previous studies, emphasising the importance of these bacteria in pneumonia diagnoses [25, 26]. For effective early-stage empirical anti-infective therapy upon hospital admission, identifying the specific bacterial infection based on patients’ underlying conditions and risk factors is crucial. This tailored approach aids in selecting appropriate antibiotics, a key measure to reduce antibiotic abuse and bacterial resistance [27]. *M. pneumoniae* detection rates in this study were significantly lower than those in Jinshan, Shanghai [6], and Kenya [28], but similar to rates observed in Hong Kong [29], indicating regional and climatic differences in bacterial prevalence. Epidemiologically, *P. aeruginosa, K. pneumoniae*, and *M. pneumoniae*, with higher positive detection rates, exhibited consistent rates across all seasons. In contrast, *S. pneumoniae* demonstrated a “double peak” prevalence in summer and winter, warranting special attention.

This study compared the detection of respiratory pathogens between 249 patients admitted to the ICU and 1017 patients admitted to the general ward. It revealed that the prevalent pathogens in the ICU were *K. pneumoniae, A. baumannii*, and *P. aeruginosa*, while the general wards showed higher occurrences of influenza virus, ADV, RV, and Chlamydia pneumoniae. This highlights the critical need for close monitoring of patients with *K. pneumoniae, A. baumannii*, and *P. aeruginosa* due to their potential susceptibility to inadequate anti-infective therapy and disease progression, potentially linked to the rise in bacterial drug resistance [30, 31]. Regarding different age groups, the paediatric patients exhibited markedly elevated positive detection rates of influenza virus (23.9%) and *M. pneumoniae* (32.6%), surpassing those observed in adult and elderly patients, a trend consistent with previous research [32]. Additionally, among adults, ADV (10.0%) and RV (6.4%) were the predominant pathogens, mostly treated in general wards with a lower likelihood of condition exacerbation. Contrastingly, the elderly, with higher risks of disease progression, exhibited major pathogens such as Klebsiella pneumoniae (11.8%) and Acinetobacter baumannii (7.1%), often leading to ICU admission for treatment. It also reflects that elderly patients with chronic cardiovascular diseases and immunodeficiencies are likely to have exacerbation of conditions and even life-threatening conditions once SARI occurs, adding substantial burdens to families and society [33, 34].

This study has several limitations. First, it is a single-centre study with a small sample size. Despite the centre’s status as the largest hospital in Central China, the generalisability of these findings is relatively limited. Second, there are fewer paediatric patients and the visitation years, potentially introducing biases into the results. Third, the study focuses solely on common pathogens, overlooking the detection of uncommon ones such as Mycobacterium tuberculosis, fungi, and Pneumocystis jirovecii. Consequently, patients with unidentified pathogens might test positive for other unidentified pathogens. Lastly, the exclusion of patients with COVID-19 from this study is notable. Given, COVID-19’s prevalence as a common respiratory virus, it needs special attention in the diagnosis and treatment processes.

## Conclusion

This study pioneers the surveillance of respiratory pathogens and epidemiological characteristics of patients with SARI in Central China, demonstrating the proliferation of various viruses and bacteria influenced by demographic factors and climate dynamics. It underscores the importance of continuous sentinel surveillance for SARI at the national and local levels. This surveillance offers a precise evaluation of SARI-associated pathogen prevalence and disease burden, crucially informing policy strategies for SARI prevention, control, and clinical management.

## Data Availability

The datasets used and/or analysed during the current study available from the corresponding author on reasonable request.

## References

[1] Liu L, Johnson HL, Cousens S, Perin J, Scott S, Lawn JE, Rudan I, Campbell H, Cibulskis R, Li M (2012). Global, regional, and national causes of child mortality: an updated systematic analysis for 2010 with time trends since 2000. Lancet.

[2] Rudan I, El Arifeen S, Bhutta ZA, Black RE, Brooks A, Chan KY, Chopra M, Duke T, Marsh D, Pio A (2011). Setting research priorities to reduce global mortality from childhood pneumonia by 2015. PLoS Med.

[3] Hodinka RL. Respiratory RNA viruses. Microbiol Spectr 2016, 4(4). 10.1128/microbiolspec.DMIH2-0028-2016 27726802

[4] Zhao Y, Lu R, Shen J, Xie Z, Liu G, Tan W (2019). Comparison Viral Epidemiol Profiles Hospitalized Child Severe Acute Respiratory Infect Beijing Shanghai China BMC Infect Dis.

[5] Wan L, Li L, Zhang H, Liu C, Li R, Wu X, Chen J (2023). Chang Pattern Common Respiratory Viruses among Child 2018 2021 Wuhan China Arch Virol.

[6] Li J, Song CL, Wang T, Ye YL, Du JR, Li SH, Zhu JM (2021). Etiological Epidemiol Characteristics Severe Acute Respiratory Infect Caused Multiple Viruses Mycoplasma pneumoniae Adult Patients Jinshan Shanghai: Pilot hospital-based Surveillance Study PLoS One.

[7] Bakaletz LO (2017). Viral-bacterial co-infections Respiratory Tract Curr Opin Microbiol.

[8] Hanada S, Pirzadeh M, Carver KY, Deng JC (2018). Respiratory Viral Infection-Induced Microbiome Alterations Secondary Bacterial Pneumonia Front Immunol.

[9] Azoulay E, Russell L, Van de Louw A, Metaxa V, Bauer P, Povoa P, Montero JG, Loeches IM, Mehta S, Puxty K (2020). Diagnosis of severe respiratory infections in immunocompromised patients. Intensive Care Med.

[10] Juliana AE, Tang MJ, Kemps L, Noort AC, Hermelijn S, Plötz FB, Zonneveld R, Wilschut JC (2021). Viral Causes Severe Acute Respiratory Infect Hospitalized Child Association Outcomes: two-year Prospective Surveillance Study Suriname PLoS One.

[11] Sharma A, Ahmad Farouk I, Lal SK. COVID-19: A Review on the Novel Coronavirus Disease Evolution, Transmission, Detection, Control and Prevention. Viruses 2021, 13(2). 10.3390/v13020202 PMC7911532 33572857

[12] Berger M, Geng B, Cameron DW, Murphy LM, Schulman ES (2017). Prim Immune Defic Dis as Unrecognized Causes Chronic Respiratory Disease Respir Med.

[13] Chakhunashvili G, Wagner AL, Power LE, Janusz CB, Machablishvili A, Karseladze I, Tarkhan-Mouravi O, Zakhashvili K, Imnadze P, Gray GC (2018). Severe Acute Respiratory infection (SARI) sentinel surveillance in the country of Georgia, 2015–2017. PLoS ONE.

[14] Pradhan M, Shah K, Alexander A, Ajazuddin, Minz S, Singh MR, Singh D, Yadav K, Chauhan NS (2022). COVID-19: clinical presentation and detection methods. J Immunoass Immunochem.

[15] Maniruzzaman M, Islam MM, Ali MH, Mukerjee N, Maitra S, Kamal MA, Ghosh A, Castrosanto MA, Alexiou A, Ashraf GM (2022). COVID-19 diagnostic methods in developing countries. Environ Sci Pollut Res Int.

[16] Guo J, Ge J, Guo Y (2022). Recent advances in methods for the diagnosis of Corona Virus Disease 2019. J Clin Lab Anal.

[17] Chen S, Kang Y, Li D, Li Z (2022). Diagn Perform Metagenomic next-generation Sequencing Detect Pathogens Bronchoalveolar Lavage Fluid Patients Pulmonary Infections: Syst Rev meta-analysis Int J Infect Dis.

[18] Jin X, Li J, Shao M, Lv X, Ji N, Zhu Y, Huang M, Yu F, Zhang C, Xie L, et al. Improving Suspected Pulmonary Infect Diagnosis Bronchoalveolar Lavage Fluid Metagenomic Next-Generation Sequencing: Multicenter Retrospective Study Microbiol Spectr. 2022;10(4):e0247321. 10.1128/spectrum.02473-21 PMC9431624 35943274

[19] Yang A, Chen C, Hu Y, Zheng G, Chen P, Xie Z, Fan H, Sun Y, Wu P, Jiang W (2022). Application Metagenomic Next-Generation Sequencing (mNGS) Using Bronchoalveolar Lavage Fluid (BALF) Diagnosing Pneumonia Child Microbiol Spectr.

[20] Yi S, Zhang WX, Zhou YG, Wang XR, Du J, Hu XW, Lu QB (2023). Epidemiol Change Influenza Virus Hospitalized Child Acute Respiratory Tract Infect Dur 2014–2022 Hubei Province China Virol J.

[21] Yu H, Huang J, Huai Y, Guan X, Klena J, Liu S, Peng Y, Yang H, Luo J, Zheng J (2014). Substantial Hospitalization Burd Influenza Cent China: Surveillance Severe Acute Respiratory Infect Influenza Viruses 2010–2012 Influenza Other Respir Viruses.

[22] Huo X, Qin Y, Qi X, Zu R, Tang F, Li L, Hu Z, Zhu F (2012). Surveillance 16 Respiratory Viruses Patients influenza-like Illn Nanjing China J Med Virol.

[23] Liu YN, Zhang YF, Xu Q, Qiu Y, Lu QB, Wang T, Zhang XA, Lin SH, Lv CL, Jiang BG (2023). Infect co-infection Patterns community-acquired Pneumonia Patients Different ages China 2009 2020: Natl Surveillance Study Lancet Microbe.

[24] Oliva J, Terrier O. Viral and Bacterial Co-Infections in the Lungs: Dangerous Liaisons. Viruses 2021, 13(9). 10.3390/v13091725 PMC8472850 34578306

[25] Rider AC, Frazee BW (2018). Community-Acquired Pneumonia. Emerg Med Clin North Am.

[26] Martin-Loeches I, Rodriguez AH, Torres A (2018). New Guidelines hospital-acquired pneumonia/ventilator-associated Pneumonia: USA vs Europe Curr Opin Crit Care.

[27] Huemer M, Mairpady Shambat S, Brugger SD, Zinkernagel AS (2020). Antibiotic Resist persistence-Implications Hum Health Treat Perspect EMBO Rep.

[28] Breiman RF, Cosmas L, Njenga M, Williamson J, Mott JA, Katz MA, Erdman DD, Schneider E, Oberste M, Neatherlin JC (2015). Severe acute respiratory infection in children in a densely populated urban slum in Kenya, 2007–2011. BMC Infect Dis.

[29] Lui G, Ip M, Lee N, Rainer TH, Man SY, Cockram CS, Antonio GE, Ng MH, Chan MH, Chau SS (2009). Role of ‘atypical pathogens’ among adult hospitalized patients with community-acquired pneumonia. Respirology.

[30] Peyrani P, Mandell L, Torres A, Tillotson GS (2019). The burden of community-acquired bacterial pneumonia in the era of antibiotic resistance. Expert Rev Respir Med.

[31] Ho J, Ip M (2019). Antibiotic-resistant community-acquired bacterial pneumonia. Infect Dis Clin North Am.

[32] Chen Y, Mah MG, Low JGH, Ooi EE, Su YCF, Moorthy M, Smith GJD, Linster M (2021). Etiology of febrile respiratory infections in the general adult population in Singapore, 2007–2013. Heliyon.

[33] Kwon KT (2023). Immune Responses Breakthr Infections after COVID-19 Vaccination J Korean Med Sci.

[34] Langerbeins P, Eichhorst B (2021). Immune Dysfunct Patients Chronic Lymphocytic Leuk Challenges Dur COVID-19 Pandemic Acta Haematol.

